# Fermi-level-managed multi-barrier heterojunction diodes for terahertz detection

**DOI:** 10.1038/s41598-025-05299-0

**Published:** 2025-07-01

**Authors:** Iñigo Belio-Apaolaza, James Seddon, Cyril C. Renaud

**Affiliations:** Department of Electronic and Electrical Engineering, University College London, London, UK

**Keywords:** Electrical and electronic engineering, Electronic and spintronic devices, Semiconductors

## Abstract

Terahertz heterodyne receivers are essential for enabling coherent, high-sensitivity signal detection. At room temperature, GaAs Schottky barrier diodes remain the leading technology but present limitations, particularly in terms of high local oscillator power requirements and contact reproducibility. The fermi-level-managed barrier diode (FMBD), an all-semiconductor InGaAs/InP heterobarrier diode originally conceived for direct THz detection, has the potential to overcome these challenges. However, the intrinsic performance of the FMBD as a frequency mixer has not been fully reported, and there has been little analysis of how to optimise its epitaxial structure for heterodyne detection. In this study, we implement a semiconductor model to predict the nonlinear IV and CV characteristics of the FMBD based on its epitaxial layers, and conduct harmonic balance simulations to extract its intrinsic conversion loss and noise temperature. The results provide a guide for designing FMBD-based THz mixers, depending on the operating frequency, available LO power, and other design factors. In addition, we introduce a novel device concept: the fermi-level-managed multi-barrier diode (FMMBD), which consists of multiple concatenated heterobarriers. This design mitigates the trade-off between device area and junction capacitance. Simulations indicate the FMMBD improves device sensitivity, with intrinsic simulated noise temperatures approaching 10$\times$ the quantum limit.

## Introduction

Terahertz (THz) detectors often deal with the reception of very weak incoming signals, which applies for both active and passive systems. This is the case for instance in radio-astronomy when looking for emission and absorption lines of the interstellar medium^1–3^, or in communications when receiving highly-attenuated wireless data signals^4–6^. Therefore, the development of high-sensitivity detectors is crucial to enable these and other terahertz applications.

Signal detectors can be classified in general into direct or heterodyne categories. While direct detection is interesting because of its low complexity, heterodyne detectors offer three main advantages: (1) highly-improved sensitivity, (2) high spectral resolution, and (3) intermediate-frequency processing^7^. Therefore, these detectors are preferred or needed in many terahertz applications. The most sensitive currently existing heterodyne detectors are based on cryogenically cooled superconductor-insulator-superconductor (SIS), and hot-electron bolometer (HEB) mixers. However, the cost, weight, and power consumption of cryogenic systems somewhat limit their use. At room-temperature, the dominant solution is still today represented by metal-semiconductor rectifying diodes, i.e. Schottky diode mixers^8–10^ due to their excellent frequency response and conversion efficiency up to a few THz, low weight and compactness, and maturity. Other technologies worth mentioning used for terahertz heterodyne detectors are: CMOS-based mixers^11,12^, field-effect-transistors (FETs) either based on III-V materials^13,14^ or graphene^15,16^, and GaAs/InGaAs photoconductive antennas^17–20^.

In terahertz Schottky mixer diodes, the rectifying contact is typically based on a moderately doped n-GaAs layer. This results in a high conversion efficiency, but also requires significant local oscillator (LO) power (over 1 mW) due to the considerable barrier height ($\sim$ 0.9 eV). Alternatively, InGaAs can be used for contact to reduce the barrier height ($\sim$ 0.22 eV) and therefore significantly reduce required LO power, at the expense of a moderate conversion efficiency detriment of approximately 2-4 dB^21–23^. This relaxation in power requirements is important to develop mixers at higher frequencies and to enable the use of LO sources limited in power, e.g. photomixers^24,25^. In any case, metal-semiconductor rectifying contacts encounter two intrinsic limitations: (1) the barrier height cannot be controlled or further reduced without a lattice mismatch, and (2) the effect of surface states compromises the reproducibility of the contact.

To address these limitations, Ito and Ishibashi^26,27^ have proposed an all-semiconductor rectifying diode based on an n-type InGaAs-InP heterojunction for terahertz detection, named fermi-level-managed barrier diode (FMBD). By adjusting the doping of the InGaAs layer at the heterointerface, the barrier height can be reduced by shifting the Fermi-level above the conduction band. This concept was previously introduced by Kleinsasser et al.^28^, where a low-barrier ($\sim$ 127 meV) InGaAs-GaAs heterojunction diode was demonstrated. Other examples of barrier-tuneable all-semiconductor rectifying diodes are AlGaAs-GaAs^29–31^, ErAs on InAlGaAs^32^, and low-barrier InGaAs-InAlGaAs^33^ heterostructures. The FMBD was initially demonstrated as a zero-bias terahertz detector by adjusting the barrier height to about 70 meV, achieving a voltage responsivity of 1110 V/W, and a noise-equivalent-power (NEP) of 3.0 $\text {pW/Hz}^{-1/2}$ at 300 GHz^34^. A considerable amount of work has followed using the FMBD as a heterodyne receiver and exploiting it mainly in terahertz communications demonstrations^35–41^. We can highlight achieved NEPs and LO powers at 300 GHz as low as 1.1$\times 10^{-18}\,\text {W/Hz}^{-1/2}$ with 6$\mu$W for a fundamental mixer^35^, 4$\times 10^{-19}\,\text {W/Hz}^{-1/2}$ with 160$\mu$W for a subharmonic mixer^37^, and 2$\times 10^{-19}\,\text {W/Hz}^{-1/2}$ with 400$\upmu$W for a balanced mixer^38^.

Despite the significant development of FMBDs as terahertz heterodyne detectors, there are still two open questions. Firstly, when considering a terahertz frequency mixer, the figures of merit of main interest are the conversion loss (CL) and noise temperature (NT). These two parameters are intrinsic to the device and allow for a fair comparison with other technologies. However, in previous FMBD works, these are not specified but rather NEP and intermediate-frequency (IF) power, while using the FMBD with a trans-impedance amplifier (TIA). The extraction of intrinsic CL and NT from published data is therefore not possible. Therefore, a study of achievable CL and NT with FMBDs is still missing. Secondly, using an all-semiconductor-based device allows for greater design flexibility to achieve the best performance for specific uses. Previous work has focused on optimizing FMBD epitaxy for zero-bias detection, not heterodyne detection. Further study is required to optimise the epitaxial structure for this purpose.

In this work, we address these questions by first implementing a semiconductor model to predict the electrical characteristics of the FMBD based on its epitaxial structure. This model is then used in harmonic balance simulations, employing the FMBD as a fundamental frequency mixer. These simulations allow us to determine the intrinsic conversion loss and noise temperature of the device, enabling us to adjust the epitaxy for optimum performance. The second contribution of this work is the proposal of a new device based on the standard FMBD epitaxy, which incorporates concatenated heterojunctions. This design reduces the equivalent intrinsic capacitance linearly with the number of heterojunctions. Consequently, it eases the requirement for smaller junction areas at higher frequencies to decrease capacitance. We show that larger area devices can thus perform equally or better than smaller ones at high frequencies, significantly alleviating the fabrication challenges associated with very small junction areas. In addition, the conversion loss to noise temperature ratio is predicted to improve, showing a sensitivity potentially better than state-of-the-art room-temperature THz receivers. This approach therefore could improve fabrication yield, reliability and performance of room-temperature THz heterodyne detectors. We name this new device fermi-level managed multi-barrier diode (FMMBD).

## Methods

### FMBD and FMMBD epitaxial structure

**Fig. 1:**
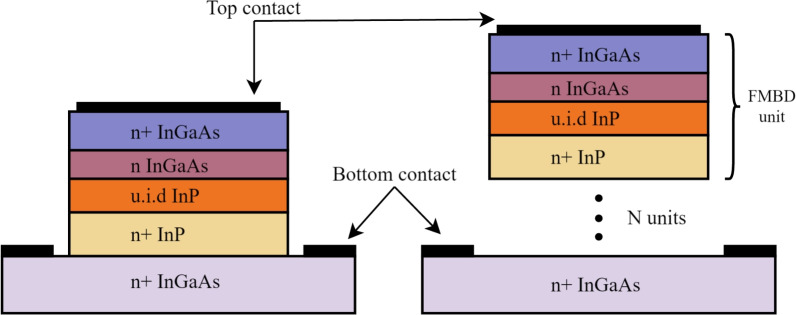
Illustration of the FMBD and FMMBD structures.

The standard FMBD epitaxy^34^ is detailed in Table 1. It comprises a lattice-matched InGaAs-InP heterojunction where the barrier height is modulated by adjusting the doping of the barrier layer (n-InGaAs). Top and bottom layers consist of highly-doped InGaAs to make low-resistance Ohmic contacts. An additional n+ InP layer is needed to avoid an electrical potential discontinuity between InP and InGaAs. The adjustment of the barrier layer doping shifts the Fermi level above the conduction band, allowing continuous barrier tuning from 0 eV to the InGaAs-InP conduction band discontinuity. In initial FMBD works^34^ the barrier layer doping was set to $5\times 10^{18} \textrm{cm}^{-3}$, resulting in a barrier height of approximately 70 meV. This doping level is the primary design parameter to adjust in the FMBD, and it is the focus of this study. In this sense, we investigate doping levels from $10^{16}$ to $10^{19}\, \textrm{cm}^{-3}$ and analyze the resulting characteristics in heterodyne detection. A doping level of $10^{16} \, \textrm{cm}^{-3}$ is set as the minimum achievable as per unintentional doping considering molecular beam epitaxy (MBE) growth^42^. Figure 1 depicts the vertical structure of the FMBD with top and bottom Ohmic contacts. In our proposed solution, the FMBD unit, which is formed by the first four layers, is repeated N times. Here, N represents the number of barriers in the FMMBD as illustrated in Fig. 1.

**Table 1:** Epitaxial structure of the FMBD with adjustable barrier height. u.i.d: unintentionally doped.

Layer function	Material	Doping (cm$^{-3}$)	Thickness (nm)
Top contact	n+ InGaAs	$3 \times 10^{19}$	80
Barrier	n InGaAs	$10^{16}$ to $10^{19}$	40
Interface	n InP	u.i.d	50
Buffer	n+ InP	$1 \times 10^{19}$	100
Bottom contact	n+ InGaAs	$3 \times 10^{19}$	600

### Semiconductor model

Based on the FMBD epitaxial structure, a model utilizing COMSOL Multiphysics Semiconductor Module is implemented. This software is a technology computer-aided design (TCAD) tool comprising a physics-based model with the drift-diffusion (DD) equations for carrier transport^43^. Since the FMBD is a majority-carrier dominated device, only electron transport is addressed in the model. First, correctly defining the semiconductor material parameters is essential to obtain an accurate model. The characteristics of the FMBD are inherently determined by the barrier height for a given barrier layer doping, making accurate determination of the barrier height essential.

In previous studies^34^, the doping level to adjust the barrier height was selected relying on photoluminiscence (PL) measurements^44^ monitoring the PL peak shift for different doping levels. However, a PL measurement is not only influenced by fermi-level shift but also bandgap narrowing and reabsorption^45^. Here, our approach is to determine the barrier height fully based in physical modelling with reasonable assumptions. The material parameters and models crucial for determining the barrier height are detailed in the Supplementary Information.

**Fig. 2:**
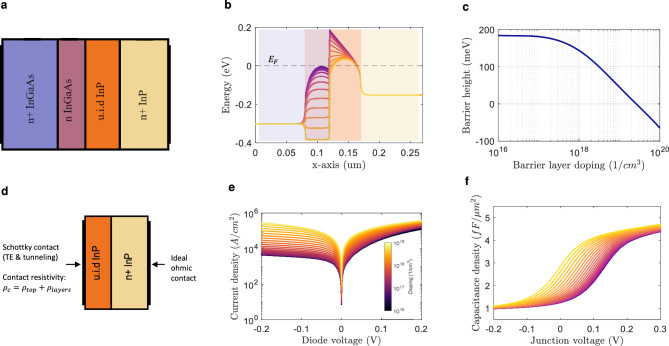
Summary of the semiconductor model process: (**a**) 1D FMBD unit structure considered in the model’s first step. (**b**) Resulting conduction band profile for doping levels from $10^{16}$ to $10^{20}$ $\textrm{cm}^{-3}$. (**c**) Resulting barrier height. (**d**) 1D InP structure considered in the model’s second step. (**e**) Resulting current density versus diode voltage. (**f**) Resulting junction capacitance density versus junction voltage.

After carefully addressing the model parameters, the energy band diagram can be solved and therefore the barrier height for a particular barrier layer doping can be determined. A summary of the semiconductor model’s first step is presented in Fig. 2. For this first calculation, a 1D model is built representing the FMBD unit as shown in Fig. 2a. The resulting conduction band profile for doping levels from $10^{16}$ to $10^{20}$ $\textrm{cm}^{-3}$ is displayed in Fig. 2b, and the barrier height in Fig. 2c.

The maximum barrier height is 187 meV for unintentially doped (u.i.d) InGaAs, and becomes 0 meV for a doping of approximately $2.82\times 10^{19}$ $\textrm{cm}^{-3}$. Note that the maximum barrier height is less than the conduction band discontinuity (210 meV, following the rule $\Delta E_c = 0.39\Delta E_g$^46^). This occurs due to band bending caused by the adjacent highly-doped InGaAs top contact layer. In the same way, this influences the fermi-level ($E_F$) to be above the conduction band even at low doping levels. In metal-semiconductor diodes, a well-known phenomenon that affects the peak barrier height is the image-force barrier lowering effect. We add this effect to correct the predicted barrier height (more details are available in the Supplementary Information). For a barrier layer doping of $5\times 10^{18}$ $\textrm{cm}^{-3}$, the peak barrier height is 87 and 66 meV before and after image-force correction, respectively.

#### Diode current and capacitance

Once the energy diagram of the FMBD unit is solved, the next and final step in the semiconductor modelling process is to extract the device’s electrical characteristics, namely current-voltage (IV) and capacitance-voltage (CV) curves. To do this, the energy diagram result of the previous step is crucial. In a heterojunction diode, the current is dominated by thermionic emission and tunneling mechanisms^47^. The TCAD tool used in this study includes a model based in these processes^43,47^. However, the model is only valid for non-degenerate semiconductors as it is based in Maxwell-Boltzmann (MB) formulation. Based on the energy diagram (see Fig. 2b), we see that $E_F$ is above the conduction band in all cases, rendering the model inapplicable. Nevertheless, the left side of the heterointerface can be treated as a degenerate semiconductor, and the current through the junction can be approximated using a metal-semiconductor thermionic emission model, i.e. a Schottky contact model^48^. The TCAD tool allows to set such a boundary condition only in metal-semiconductor interfaces. Therefore, the model is simplified by including only the InP layers (see Fig. 2d), where the left and right contacts are of Schottky and ohmic type respectively. The Schottky model requires the Richardson constant ($A^{*}$) as input, which is related to the electron effective mass. Here, we consider the effective mass of the InP interface layer, which leads to a Richardson constant of $9.62\ \mathrm{A/(cm}^2 \textrm{K}^2)$. In addition to thermionic emission, tunneling across the barrier has a significant impact on the diode current, specially at low barriers. This mechanism is added with the Wentzel–Kramers–Brillouin (WKB) 1D approximation^49^.

Regarding the InGaAs top layers, these contribute to a fixed series resistivity ($\rho$) which is extracted from the low-field mobility model (see Supplementary Information). In addition, the Ohmic contact resistivity must be considered. Although the FMBD has both top and bottom Ohmic contacts, the top contact is the limiting factor in terms of area, while the resistance of the bottom contact can be considered negligible. The contact resistivity is influenced by a variety of factors, including the metallization scheme and the contact material. Additionally, it is significantly affected by the fabrication processes. Consequently, experimental determination of the contact resistivity is necessary. In this study, we extract the contact resistivity from the IV curves reported in two published works^27,34^. These two works achieve a contact resistivity of approximately $44$ and $20\Omega \upmu {\rm m}^2$. Following all previous considerations, the FMBD IV curve can be calculated. In Fig. 2e the resulting current density curves (JV) for different doping levels with a contact resistivity of $44\Omega \, \upmu \textrm{m}^2$ are shown. The remaining CV characteristic of the diode is obtained by applying a small-signal excitation to the structure and measuring the imaginary part of the current. The resulting junction capacitance density for different doping levels is displayed in Fig. 2f.

#### Model validation

**Fig. 3:**
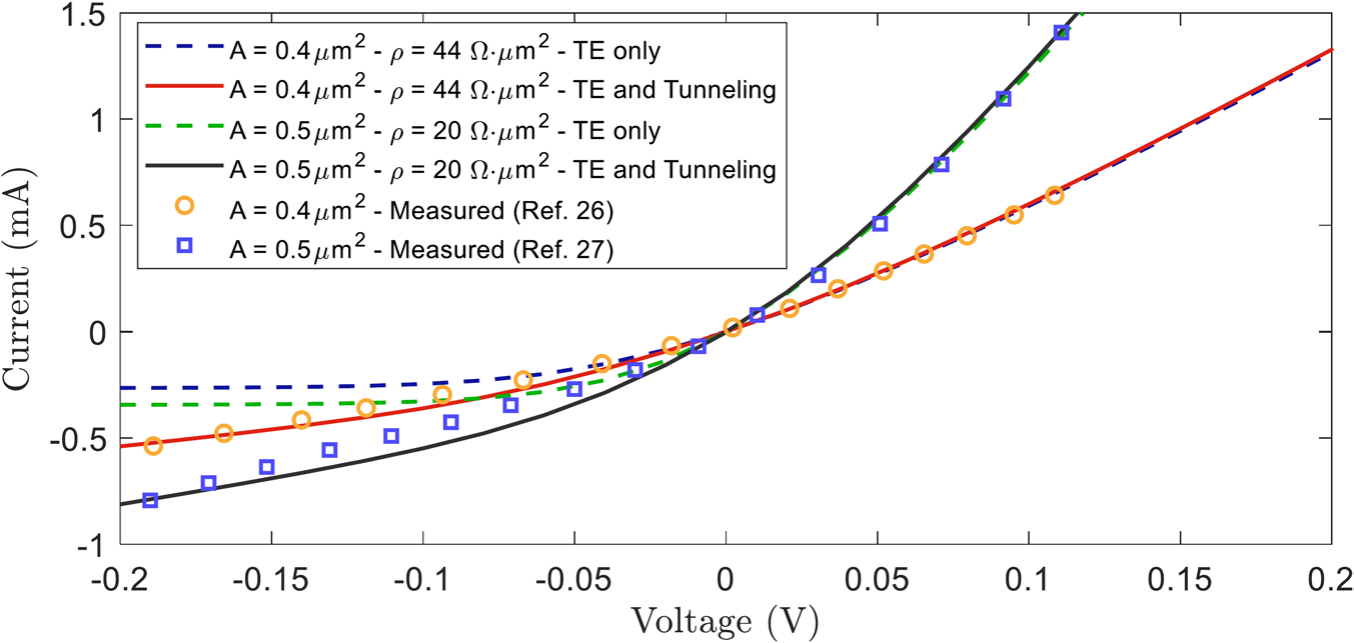
Current-voltage curves comparison for 0.4 and 0.5 $\upmu \textrm{m}^2$ FMBDs with a barrier layer doping of $5 \times 10^{18} \textrm{cm}^{-3}$. TE: Thermionic Emission.

To verify the described modelling approach, the measured electrical characteristics from previous works^27,34^ can be compared to the predicted ones. We do this for two FMBDs with a barrier layer doping of $5 \times 10^{18} \textrm{cm}^{-3}$ and diode areas of $0.4$ and $0.5 \upmu \textrm{m}^2$. The measured barrier height in these works, which results from fitting the IV curve to a thermionic current model, is approximately 70 meV. In comparison, our model estimates a barrier height of 66 meV. The measured IV curves and the predicted by the model are displayed in Fig. 3. These results highlight the importance of incorporating tunneling effects. When only thermionic emission is considered, the reverse bias voltage remains constant, which fails to match the measured curves. However, with the inclusion of tunneling, the predicted and measured curves show strong agreement. Some moderate discrepancies at reverse bias can be attributed to the model approximations and non-idealities.

### Circuit model

To analyze the performance of the FMBD as a heterodyne detector, an equivalent circuit that reflects the electrical characteristics of the diode must be implemented. We implement such a circuit including IV and CV characteristics, as well as thermal and Shot noise sources. The circuit is depicted in Supplementary Figure 1, and described in detail in the Supplementary Information, with the resulting fitted parameters depicted in Supplementary Figure 2.

### Harmonic balance simulations

**Fig. 4:**
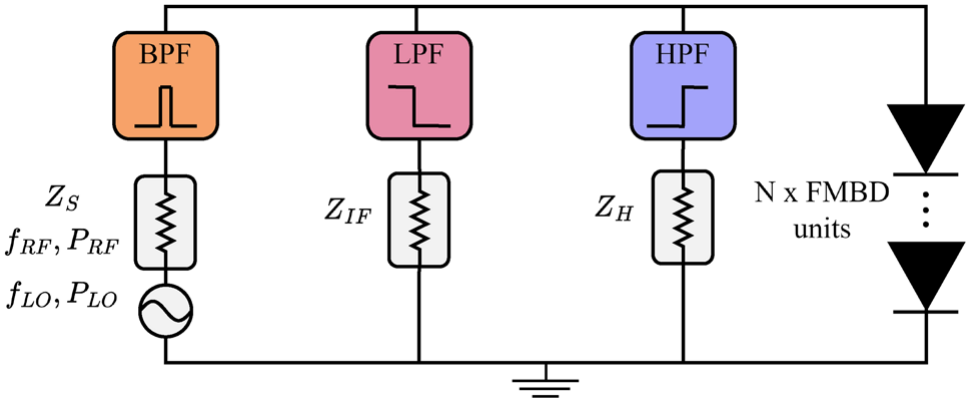
Single-ended mixer circuit for harmonic balance simulations. BPF: Bandpass filter, LPF: Low-pass filter, HPF: High-pass filter.

To evaluate performance as a frequency mixer, we conduct harmonic balance simulations that account for the non-linear IV and CV characteristics of the equivalent circuit. These simulations are performed using Advanced Design System (ADS), a standard software tool for mixer analysis. We implement a single-ended frequency mixer circuit, depicted in Fig. 4. A custom component is created to implement the equivalent circuit of Supplementary Figure 1. To study the FMMBD, a number $N$ of these elements are concatenated, where $N$ is the number of barriers, and we study the performance of the FMMBD up to 4 barriers. Another important aspect is the impedance associated to these frequency components. The output IF impedance is set to 50 $\Omega$ as this is the standard for other IF components like low-noise amplifiers (LNAs). The source impedance, which includes RF and LO signals, is optimised for each specific case, consisting of device area, operating frequency, barrier layer doping, contact resistance, and number of barriers. The impedance associated to higher harmonics, which was found to have a minor effect in performance, is set to 1000 $\Omega$. We investigate the down-conversion efficiency at radio frequencies (RF) ranging from 100 to 2000 GHz, setting the LO frequencies so that the IF frequency lies at 1 GHz. Ideal filter blocks are used to separate RF, LO, IF and higher-harmonic signals. The RF power is set to $-50$ dBm, and the LO power is varied from $-30$ to 0 dBm. These simulations allows us to benchmark the FMBD and FMMBD as a fundamental single-ended mixer, extracting the intrinsic down-conversion capabilities in terms of conversion loss and noise temperature.

## Results

### Single barrier

**Fig. 5:**
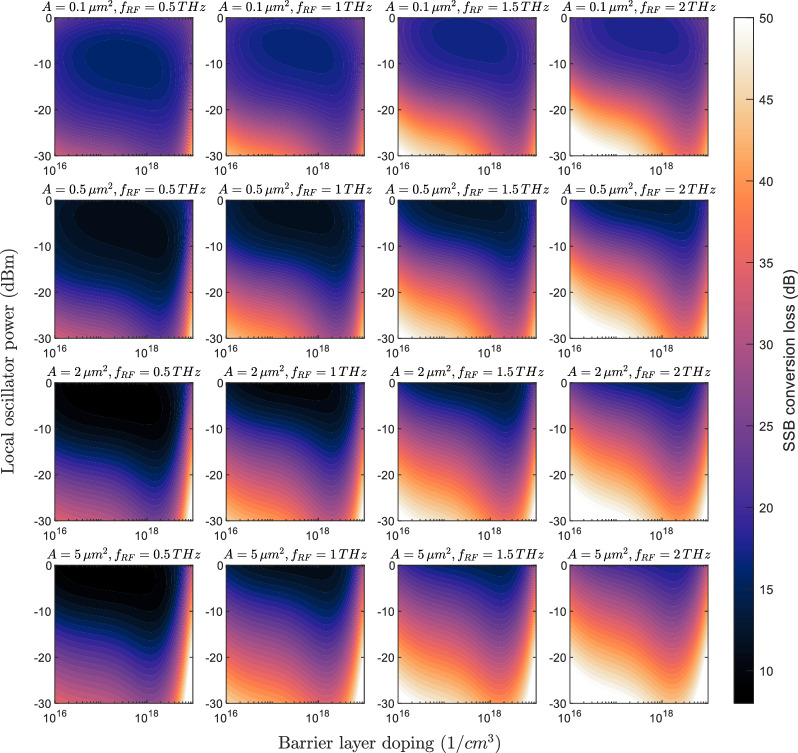
2D contour plots of simulated FMBD conversion loss as a function of barrier layer doping and local oscillator power. Results are presented for RF frequencies of 0.5, 1, 1.5, and 2 THz, with device areas of 0.1, 0.5, 2, and 5 $\upmu m^2$, and a contact resistivity of $20\Omega \, \upmu m^2$.

To begin with, we focus on evaluating the intrinsic performance of the FMBD, being this the first goal of the study. In Fig. 5, 2D conversion loss maps are shown for RF frequencies of 0.5, 1, 1.5, and 2 THz, with device areas of 0.1, 0.5, 2, and 5 $\upmu m^2$, and a contact resistivity of $20\Omega \mu m^2$. This and the rest of maps in this study are shown for barrier layer doping between $10^{16}$ and $10^{19} \textrm{cm}^{-3}$ and LO powers between $-30$ and 0 dBm. The first noticeable difference is in the optimum LO power and doping. As the device area increases, higher power is required to achieve optimum conversion efficiency. Similarly, as the RF frequency increases, more LO power is needed. Both factors are directly related to the device’s cut-off frequency, which is primarily influenced by the junction capacitance. In this sense, the optimum LO power can be as low as -15 dBm for ${0.1}\,{\upmu }\text {m}^{2}$ at 0.5 THz, and as high as $-2$dBm for ${5}\,{\upmu }\text {m}^{2}$ at 2 THz.

Regarding the barrier layer doping, a higher value means a lower barrier height and therefore the optimum LO power is reduced. The optimum doping to achieve a good trade-off between required LO power and down-conversion efficiency, is found between $1\times 10^{18}$ and $4\times 10^{18} \textrm{cm}^{-3}$. That range can be translated to a barrier height between 144 and 89 meV. A high-doping level also alleviates the penalty of insufficient LO power, and conversion loss below 30 dB can be achieved with an LO power of $-20$ dBm even at 2 THz for a doping of $3\times 10^{18} \textrm{cm}^{-3}$. In terms of minimum conversion loss, values as low as 9 and 15 dB are achieved at frequencies of 0.5 and 2 THz respectively. It is evident that the minimum conversion is lower as the area is increased, particularly at low frequencies. This occurs because even though the junction capacitance increase with area, the reduction in contact resistance compensates this effect reflecting even on a slight improvement in conversion loss. This phenomenon is more pronounced in the extreme case of ${0.1}\,{\upmu }\text {m}^{2}$ , where the contact resistance is as high as $200 \Omega$.

**Fig. 6:**
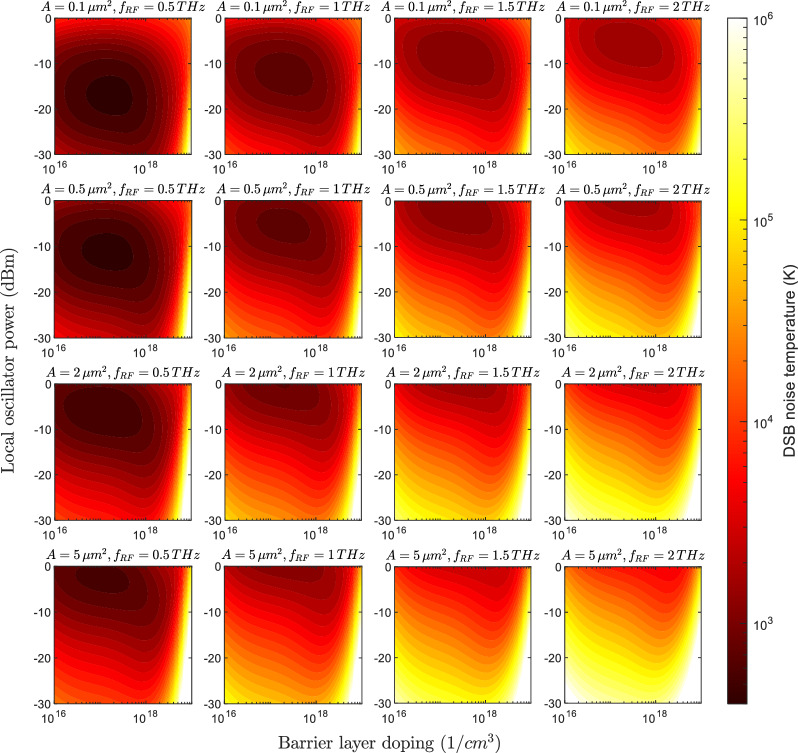
2D contour plots of simulated FMBD noise temperature as a function of barrier layer doping and local oscillator power. Results are presented for RF frequencies of 0.5, 1, 1.5, and 2 THz, with device areas of 0.1, 0.5, 2, and 5 $\upmu \textrm{m}^2$.

Double-side-band (DSB) noise temperature results are shown in Fig. 6. Similar behaviour is observed with some discrepancies. First, the optimum LO power is reduced compared to the conversion loss result, requiring for example just $-20$ dBm with an area of ${0.1}\,{\upmu }\text {m}^{2}$. The optimum doping is also reduced. Both features are attributed to Shot noise. This noise contribution is directly proportional to the DC diode current. Therefore, either for increasing LO power or decreasing barrier height the contribution is more significant, which can detriment the final noise figure. This leads to an unintuitive result of achieving the minimum noise temperature at a non-minimum conversion loss. A minimum noise temperature of 480 and 1953 K is achieved at 0.5 and 2 THz respectively.

**Fig. 7:**
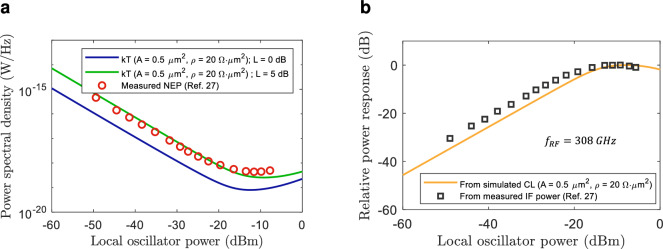
Down-conversion simulated FMBD performance comparison with experimental results: (**a**) Noise equivalent power (NEP) and $kT$ by considering no additional coupling loss (blue curve) and an excess loss of 5 dB (green curve), and (**b**) Relative power response.

As previously mentioned, direct comparison of predicted and measured performance is not possible because these figures of merit were not measured in previous works^26,27^. However, we can make an indirect comparison. In case of NEP, this can be compared to the minimum detectable signal (MDS) spectral density which can be approximated by $kT$, where $T$ is the noise temperature. That comparison is shown in Fig. 7a for a device with an area of ${0.5}\,{\upmu }\text {m}^{2}$ and a doping level of $5\times 10^{18} \textrm{cm}^{-3}$ at 308 GHz^27^. The minimum measured NEP is $4.4\times 10^{-19} \mathrm{W/Hz}$ compared to the predicted $7.98 \times 10^{-20} \mathrm{W/Hz}$ (blue curve). In both cases, this occurs for approximately $-12$ dBm of LO power. There is an offset by a factor of approximately 5.5. This can be expected because the MDS and NEP are not directly comparable as noise from other components like the TIA influences the NEP. In addition, the predicted performance does not take into consideration other factors which are relevant like coupling or circuit losses, and device parasitics. In this sense, the previous work selected for comparison^27^ is based on an FMBD packaged with a silicon lens, i.e. quasi-optical coupling, which results in additional RF coupling losses. By adding an excess 5 dB coupling loss to our simulations, the MDS strongly matches the measured NEP, further validating the modelling process. This added loss accounts not only for the RF coupling loss of the receiver, but also for other non-idealities, including circuit parasitics and high-frequency phenomena reducing the conversion efficiency^50^. An additional comparison can be done in terms of down-converted power response with respect to LO power. This is shown in Fig. 7b. Similarly, the predicted response and saturation point matches the measured one.

### Multiple barriers

**Fig. 8:**
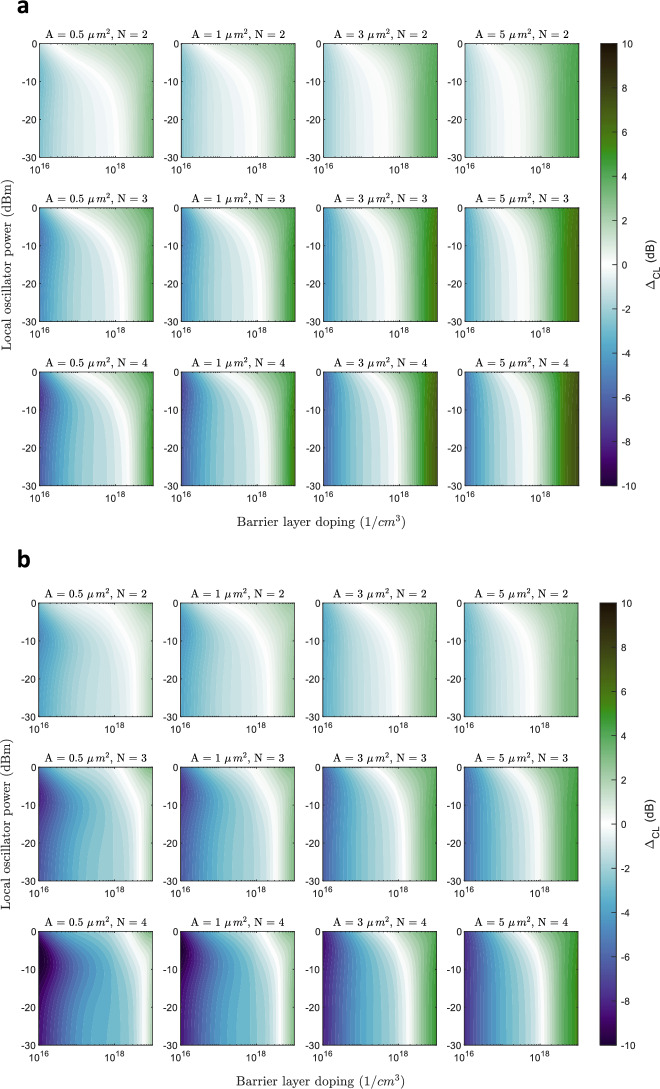
2D contour plots of simulated conversion loss difference ($\Delta _{CL}$) when employing multiple barriers (FMMBD) with a contact resistivity of (**a**) $44\Omega \,\upmu \textrm{m}^2$ and (**b**) $20\Omega \, \upmu \textrm{m}^2$. Results are shown for up to 4 barriers for device areas of 0.5, 1, 3, and 5 $\upmu \, \textrm{m}^2$. The RF frequency is set to 2 THz.

The second part of the report focuses on the performance of the FMMBD with up to four barriers. We present the differences in conversion loss and noise temperature, expressed as noise figure differences in dB, for devices with two, three, and four barriers. In the previous section, the performance was evaluated for the lowest reported contact resistance ($\simeq 20 \Omega \,\upmu m^2$) to assess the potential of the FMBD as a heterodyne receiver under optimum but realistic conditions. For a more comprehensive analysis, we also include a comparison of the FMMBD with higher contact resistance ($\simeq 44 \Omega \, \upmu m^2$) revealing interesting behaviour, as will be discussed. The conversion loss comparison is presented in Fig. 8a, b. This figure shows the difference in conversion loss taking a single-barrier FMBD as a reference at a the maximum frequency of operation considered in this study (2 THz). The first thing to note is that the conversion loss can be improved with the inclusion of multiple barriers. This occurs at relatively high-dopings, typically $1\times 10^{18} \textrm{cm}^{-3}$ and above, which is attributed to the more significant increase in the resistance of the FMMBD series attributed to the InGaAs barrier layer. The improvement in conversion loss is more pronounced with a greater number of barriers and larger area of the device. This is explained by the reduction in effective junction capacitance with the inclusion of more barriers. For small areas, the junction capacitance reduction have less impact than with large areas, because the capacitance is already notably small for a single-barrier device. This improvement is less severe when the contact resistance is lower which is related to a detriment in the non-linear IV curve characteristics when adding multiple barriers. This means that the down-conversion efficiency from a point of view of non-linear IV is somewhat worse with multiple barriers. These results highlight a trade-off between (1) series resistance, (2) junction capacitance and (3) intrinsic IV conversion efficiency.

**Fig. 9:**
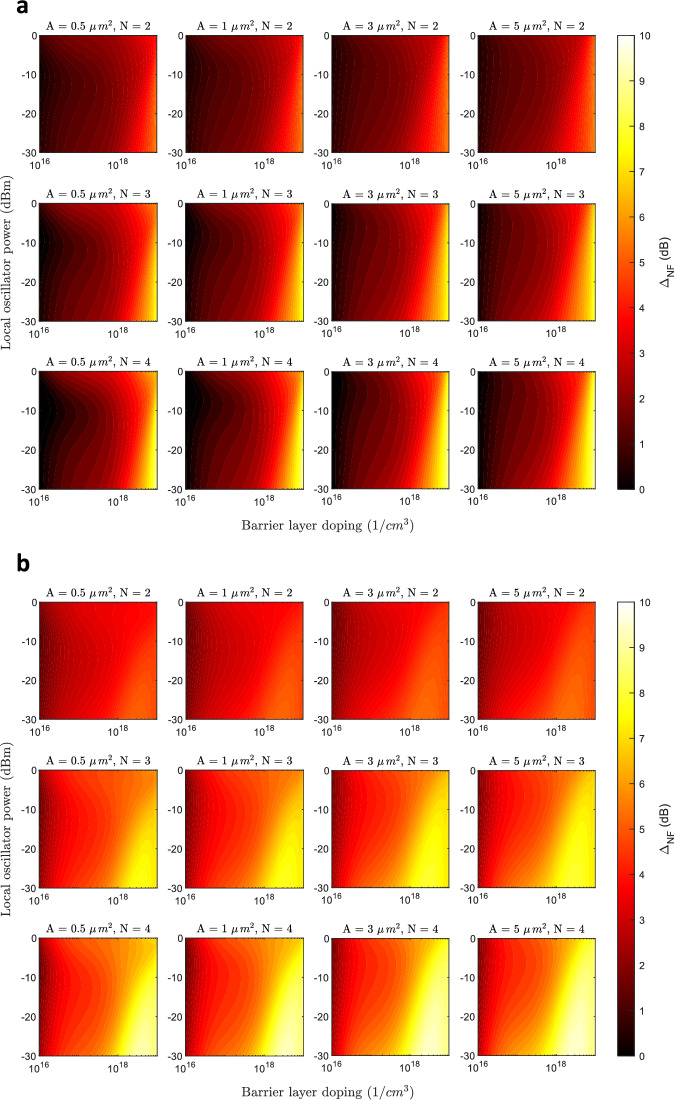
2D contour plots of simulated noise figure difference ($\Delta _{NF}$) when employing multiple barriers (FMMBD) with a contact resistivity of (**a**) $44\Omega \, \upmu \textrm{m}^2$ and (**b**) $20\Omega \, \upmu \textrm{m}^2$. Results are shown for up to 4 barriers for device areas of 0.5, 1, 3, and 5 $\upmu \textrm{m}^2$. The RF frequency is set to 2 THz.

The noise figure comparison shown in Fig. 9a, b reveals a unique behaviour. In contrast to conversion loss, the noise figure improves for almost every doping, area and number of barriers. Again, this improvement is more significant for a high contact resistance diode, achieving up to 9.62 dB of improved noise figure for an area of ${5}\,{\upmu }\text {m}^{2}$. For alternative visualization, a noise temperature and conversion loss comparison for a single doping level of $4\times 10^{18} \textrm{cm}^{-3}$ is shown in Supplementary Figure 3. This comparison shows NT and CL at 2 THz for a single-barrier and four-barrier cases, low and high contact resistance, and areas of 0.5, 2, and ${5}\,{\upmu }\text {m}^{2}$. In terms of conversion loss, for a low contact resistance the improvements are less significant, showing that for ${0.5}\,{\upmu }\text {m}^{2}$ the conversion loss is approximately equal using one or four barriers. In comparison, for a high contact resistance, the conversion loss is improved in all cases, and even an equal conversion loss is achieved with an increased area of ${2}\,{\upmu }\text {m}^{2}$ and four barriers compared to ${0.5}\,{\upmu }\text {m}^{2}$ and one barrier. However, when looking at noise temperature we see that this figure is always improved with the inclusion of four barriers, even when the conversion loss is equal. For example, with a moderate LO power of -5 dBm and a device of ${0.5}\,{\upmu }\text {m}^{2}$, a noise temperature of 1240 K is achieved with four barriers at 2 THz, in comparison to 4210 K for a single barrier. These results reveals that the NT-to-CL ratio improves with the number of barriers.

**Fig. 10:**
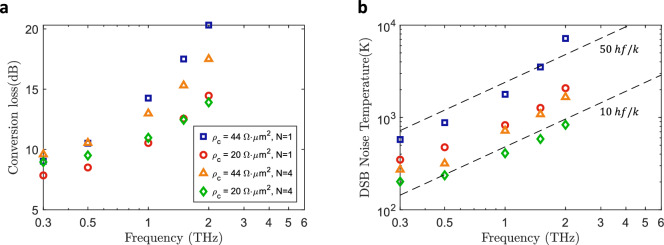
Minimum simulated conversion loss (**a**) and noise temperature (**b**) for different RF frequencies. Results are shown for contact resistivities of $44\Omega \, \upmu \textrm{m}^2$ and $20\Omega \upmu \textrm{m}^2$, including single barrier (FMBD) and 4 barrier cases (FMMBD).

To further assess performance differences when including multiple barriers, we show the minimum achieved CL and NT from 0.3 to 2 THz in Fig. 10. This includes high and low contact resistance scenarios for single and four barrier cases. Device areas as small as ${0.5}\,{\upmu }\text {m}^{2}$ are considered with LO power up to -3 dBm. As evidenced in Fig. 10a, the benefits of adding more barriers in terms of conversion loss are limited, being more significant for high contact resistance and high frequencies. A minimum conversion loss of 13.9 dB is predicted at 2 THz with four barriers. It is important to keep in mind that these results refer to the optimum scenarios keeping the area of the device reduced to ${0.5}\,{\upmu }\text {m}^{2}$. We have shown that the improvement in conversion loss with multiple barriers is more significant for greater areas. Nevertheless, the noise temperature comparison in Fig. 10b shows that the key improvement of the FMMBD lies in the sensitivity of the terahertz detection. We see that for all scenarios the noise temperature is improved. For a high contact resistance, the noise temperature follows a trend close to 50 times the quantum limit, while for the best case (low contact resistance and four barriers), the trend is close to 10 times the quantum limit, with a minimum noise temperature of 832 K at 2 THz. Interestingly, the noise temperature for an FMMBD with high contact resistance is found to be lower compared to a single barrier device with low contact resistance.

## Discussion

The results of this study provide extensive insight into the intrinsic frequency conversion capabilities of FMBDs. The minimum conversion loss and noise temperature from 0.3 to 2 THz ranges from approximately 8 to 14 dB and 350 to 1953 K. We have shown that the ideal barrier height to achieve this performance is between 150 and 90 meV, which is equivalent to a barrier layer doping level between $1 \times 10^{18}$ and $4 \times 10^{18}cm^{-3}$. To put this into perspective, the CL and NT are comparable or better than state-of-the-art GaAs Schottky mixers. For example, in a recent work^50^ a GaAs Schottky mixer was demonstrated at 2 THz achieving a record noise temperature of 4000-6000 K with all-solid-state technology. It is important to remark that our calculations do not include device parasitics and circuit losses, which have a significant effect at 2 THz. Therefore, the predicted performance is expected to degrade. However, these results highlight that the FMBD is a promising candidate to compete with classic GaAs Schottky mixer while improving the reproducibility of the diode contact due to the all-semiconductor-based barrier. In addition, we have shown how the LO power can be significantly reduced, achieving optimum performance with only $\sim {100}{\upmu }\text {W}$ at 2 THz for a device area of ${0.1}\,{\upmu }\text {m}^{2}$ in comparison to a power over 1 mW required for GaAs Schottky mixers^8,9,51^ of similar anode areas.

In the second part of this study, we have demonstrated that the use of concatenated heterobarriers to build FMMBDs improves device performance. Although the reduction in minimum achievable conversion loss is notable mainly at high contact resistances and high frequencies as evidenced in Fig. 10a, this assumes a device area as small as ${0.5}\,{\upmu }\text {m}^{2}$. The advantage of employing multiple barriers becomes more evident when larger areas are used, allowing for equivalent performance to smaller-area devices. This is a key benefit, as the fabrication of submicron devices is more challenging and negatively impacts device yield. Furthermore, larger devices exhibit superior power dissipation properties, and the use of multiple barriers reduces the maximum electric field across each barrier, enhancing power handling capabilities^52^. Nevertheless, the main advantage of the FMMBD is related to the sensitivity of the terahertz detection, i.e. noise temperature. As the NT-to-CL ratio improves with an increasing number of barriers, this results in a lower achievable noise temperature, with predictions indicating a trend approaching 10 times the quantum limit for four barriers. For comparison, this is consistent with the behaviour typically observed in cryogenically-cooled HEBs^53,54^. At 2 THz, we have shown a predicted noise temperature as low as 832 K for a four-barrier FMMBD, compared to 815 K reported for a HEB at 4.7 THz^54^. The sensitivity improvement is directly linked to a reduction in shot noise, as increasing the number of barriers reduces the average current through the device. This, combined with maintaining or enhancing the conversion loss thanks to a reduction of junction capacitance, accounts for the observed sensitivity enhancement. The use of multiple-barrier semiconductor diodes as frequency mixers was initially proposed and tested at 10 GHz in 1994^52^. In that work, the authors experimentally demonstrated that the NT-to-CL ratio improves with additional barriers, lending further confidence to our results. In principle, the same concept could be applied to classic GaAs Schottky mixers by concatenating multiple diodes. However, the increase in circuit parasitics and footprint makes this approach impractical for THz mixers.

While the results of this study are promising, our evaluation of FMBDs and FMMBDs was limited to intrinsic diode characteristics, omitting parasitics and losses associated with practical implementations. These factors are highly dependent on receiver design, which must be optimised for a targeted frequency range and determine the overall receiver performance. In contrast, our aim was to investigate a wide frequency span to characterise intrinsic frequency conversion performance, making the inclusion of these non-idealities beyond the scope of this work. Nevertheless, we have demonstrated that at 300 GHz, the minimum simulated MDS is 5.5 times lower than the measured NEP of a quasi-optically coupled FMBD receiver. Using this offset as a reference, the expected noise temperatures at 2 THz become 10742 K for a single-barrier FMBD and 4576 K for a four-barrier FMMBD. This latter value is comparable to the record-low noise temperature achieved by a GaAs Schottky mixer^50^, albeit with a larger diode area (${0.5}\,{\upmu }\text {m}^{2}$ vs. ${0.1}\,{\upmu }\text {m}^{2}$) and reduced LO power requirement (0.5 mW vs. 1.5 mW). While the 5.5$\times$ correction factor is specific to this example and would vary with receiver design, coupling scheme, and operating frequency, this simple comparison illustrates the promising prospects of FMMBD-based THz receivers.

There are several areas in this work that could be further developed. First, the semiconductor model used here could be enhanced in multiple ways. For simplicity, a Schottky barrier boundary condition was employed to calculate the thermionic current through the barrier. Implementing a more comprehensive model specifically derived for heterobarriers with degenerate semiconductors^48,55,56^ could potentially provide a better fit to the measured IV curves. Additional physical models, such as velocity saturation and heating effects, could also be incorporated. Despite these potential improvements, we have provided a validation of our modelling approach by comparing measured and predicted IV curves, and by an indirect comparison regarding measured NEP and relative power response. In terms of noise contributions, we neglected hot-electron noise in this study, which becomes significant at high current densities in the diode^57,58^. Although the FMBD/FMMBD requires low LO power, the current density is comparable to that of GaAs Schottky mixers driven with high LO power due to the low-barrier characteristic. Including this noise contribution is complex and requires detailed knowledge of specific properties dependent on the device and material type^59^. However, the degradation in noise temperature due to this factor at room temperature is expected to be moderate, as shot and thermal noise remain the dominant contributors^57^. To further reduce the noise temperature, cryogenic cooling using liquid nitrogen could be considered. This has been applied to GaAs Schottky mixers, where improvements by a factor of approximately 2.5^60^ can be achieved. Finally, this study has focused on evaluating the performance of a single-ended FMBD/FMMBD configuration. Extending it to other mixer architectures, such as balanced or anti-parallel, by using the same modelling approach would be of interest.

## Supplementary Information

Supplementary Information.

## Data Availability

All data supporting the findings of this study are available within the manuscript and the Supplementary Information.
