# Supplementary material for: Fermi-level-managed multi-barrier heterojunction diodes for terahertz detection
Source: Sci Rep. 2025 Jul 1;15:21237. doi: 10.1038/s41598-025-05299-0 (PMC12214826; doi:10.1038/s41598-025-05299-0)
Supplement: Supplementary file 1 — Supplementary Information. [file 41598_2025_5299_MOESM1_ESM.pdf]

Supplementary information for:

Fermi-level-managed multi-barrier heterojunction diodes for terahertz detection

Iñigo Belio-Apaolaza[1,*], James Seddon[1], and Cyril C. Renaud[1]

[1]Department of Electronic and Electrical Engineering, University College London, London, UK
[*]inigo.apaolaza.21@ucl.ac.uk

Supplementary Methods

Semiconductor model

Material parameters and models

To determine the barrier height in a heterointerface, the following must be carefully addressed: (1) Conduction band discontinuity, (2) Bandgap narrowing, and (3) Doping-dependent effective mass. Reports on the conduction band discontinuity in lattice-matched InGaAs-InP heterojunctions exhibit certain variability[1], ranging from 210 meV[2] to 250 meV[3]. Here we assume a discontinuity of 210 meV, following the rule $\Delta E_c = 0.39\Delta E_g$[4]. This is set by adjusting the InGaAs electron affinity (χ_s) to 4.59 eV considering 4.38 eV for InP[1] as shown in Table 1. This condition is only valid for undoped layers, and bandgap narrowing must be considered to account for the level change in the conduction band at the barrier layer. We calculate the bandgap narrowing effect with the Jain-Roulston model[5]

$$\Delta E_g = A_{BGN} \cdot N_D^{1/3} + B_{BGN} \cdot N_D^{1/4} + C_{BGN} \cdot N_D^{1/2}, \tag{1}$$

where A_{BGN}, B_{BGN} and C_{BGN} are model coefficients, and N_D is the doping concentration in cm^{-3}. The coefficients used here, displayed in Table 1, are found in previous studies[6,7]. We assume a 50:50 split between conduction and valence bands ($\alpha_{BGN} = 0.5$), which has shown reasonable agreement with experimental results[8]. Finally, doping-dependent effective electron mass (m_n^*/m_0) must be considered to determine the effective density of states in the conduction band (N_c), which in turn is related to the fermi-level position due to band-filling. Here we use a 4-th order polynomial approximation

$$m_n^*/m_0 = A_M \cdot log_{10}(N_D)^4 + B_M \cdot log_{10}(N_D)^3 + C_M \cdot log_{10}(N_D)^2 + D_M \cdot log_{10}(N_D) + E_M, \tag{2}$$

where A_M, B_M, C_M, D_M, E_M are the polynomial coefficients. The values used in this work are shown in Table 1, which are obtained by fitting measured data from previous studies[9]. To account for the variation in device resistance due to carrier transport through the layers, we implement a doping-dependent low-field mobility (μ_n) model with the following expression valid for room-temperature (300 K)[10]

$$\mu_n = \mu_{n_{min}} + \frac{\mu_{n_{max}}^{\theta_1} - \mu_{n_{min}}}{1 + \left(N_D/N_{ref}^{\theta_2}\right)^{\lambda}}, \tag{3}$$

where $\mu_{n,min}$ and $\mu_{n,max}$ are the mobility at low and very high doping concentrations respectively, N_{ref} is the doping concentration at which the mobility is $\mu_{n,max}/2$, and θ_1, θ_2, λ are additional fitting parameters. The values used in this study[10] are displayed in Table 1. The material parameters are completed with the relative static and dynamic dielectric constants (ε_s and ε_d) and bandgap energy (E_g) as shown in Table 1. Carrier recombination effects were not added to the model as their contribution to the diode current was found to be negligible compared to thermionic emission and tunneling through the barrier.

Barrier height correction

Under the uniform field approximation[11], the reduction of the peak barrier height due to image-force barrier lowering can be estimated with[12]

	Material	
**Parameter**	InGaAs	InP
ε_s	13.9	12.5
ε_d	11.6	9.6
$E_g [eV]$	0.74	1.34
$\chi_s [eV]$	4.59	4.38
m_n^*/m_0 (undoped)	0.041	0.08
$\mu_{n_{max}} [cm^2/Vs]$	14000	5200
$\mu_{n_{min}} [cm^2/Vs]$	320	400
$N_{ref} [cm^{-3}]$	$1.3 \cdot 10^{17}$	$3 \cdot 10^{17}$
λ	0.48	0.47
θ_1	1.59	2
θ_2	3.68	3.25
A_{BGN}	$4.76 \cdot 10^{-8}$	$2.25 \cdot 10^{-8}$
B_{BGN}	$9.99 \cdot 10^{-9}$	0
C_{BGN}	0	0
α_{BGN}	0.5	0.5
A_M	$1.75741 \cdot 10^{-3}$	$1.01163 \cdot 10^{-3}$
B_M	$-1.19615 \cdot 10^{-1}$	$-6.89059 \cdot 10^{-2}$
C_M	$3.05326 \cdot 10^{0}$	$1.76126 \cdot 10^{0}$
D_M	$-3.46383 \cdot 10^{1}$	$-2.01865 \cdot 10^{1}$
E_M	$1.473876 \cdot 10^{2}$	$8.54342 \cdot 10^{1}$

Supplementary Table 1: Material parameters used in the semiconductor model including doping-dependent low-field mobility, effective mass and bandgap narrowing models.

$$\Delta\phi \simeq \phi_B \pi^{-1/2} \frac{\phi_B}{\frac{e^2}{2\varepsilon_0}\left(\frac{N_D}{\varepsilon_s \varepsilon_d^2}\right)^{1/3}}, \tag{4}$$

where ε_0 is the vacuum permittivity, and ϕ_B is the barrier height obtained from the energy band calculation.

Layer resistance

The resistivity (ρ) of a bulk semiconductor layer can be calculated with

$$\rho = \frac{t}{e\mu_n N_D}, \tag{5}$$

where t is the layer thickness, e is the electron charge, μ_n is the low-field mobility, and N_D is the layer doping.

Circuit model

The base element of the equivalent circuit (Supplementary Figure 1) is a voltage-controlled current source that represents the thermionic and tunneling junction current, which in a heterojunction diode has the form[13,14]

$$I_J = I_s \left[exp\left(\frac{eV_J}{n_f kT}\right) - exp\left(\frac{-eV_J}{n_r kT}\right)\right], \tag{6}$$

where V_j is the junction voltage, n_f and n_r are the forward and reverse ideality factors, k is the Boltzmann constant, T is the temperature, and I_s is the saturation current calculated by the following equation:

$$I_s = AA^* T^2 exp\left(\frac{-e\phi_B}{kT}\right), \tag{7}$$

where A is the area of the device and ϕ_B is the barrier height. Given our focus on room-temperature operation, the temperature is fixed to 300 K. The top contact and InGaAs layers resistances are modelled by R_{s1} and its value is determined by the Ohmic contact resistivity and layer resistivity. R_{s2} represents the resistance from the InP layers and its value is obtained by fitting the simulated IV curves. The ideality factors n_f and n_r are adjusted to minimize the error between the IV curve generated by the TCAD tool and that of the circuit model. The resulting fitting parameters are shown in Supplementary Figure 2. The CV characteristics are implemented with the capacitor C_j. The following expression is used to fit the simulated curves of capacitance density.

$$C_j = A\left(A_c + \frac{B_c}{1+exp(-C_c(V_j+D_c))} + exp(F_c V_j)\right), \tag{8}$$

where A_c, B_c, C_c, D_c and F_c are fitting parameters. The resulting values after fitting are shown in Supplementary Figure 2. The circuit model is completed with thermal and shot noise sources, implemented with a current noise source. The mean squared value of the thermal noise current spectral density can be written as

$$\frac{< i_{N,T}^2 >}{\Delta f} = \frac{4kT}{R_s}, \tag{9}$$

where R_s is the series resistance associated with thermal noise. In case of the FMBD, R_{s1} and R_{s2} are responsible for thermal noise. The shot noise current spectral density can be modelled as

$$\frac{< i_{N,S}^2 >}{\Delta f} = 2eI_d, \tag{10}$$

where I_d is the average current through the diode. Here, we assume the Shot noise from each FMMBD barrier to be uncorrelated. Therefore, the noise contribution from each barrier is added independently.

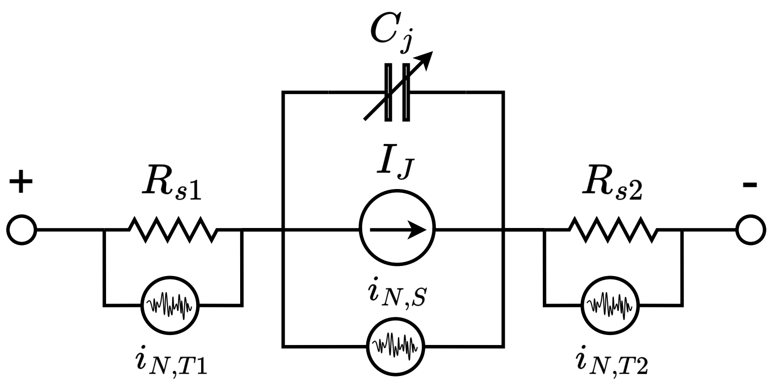

Supplementary Figure 1: FMBD equivalent circuit model.

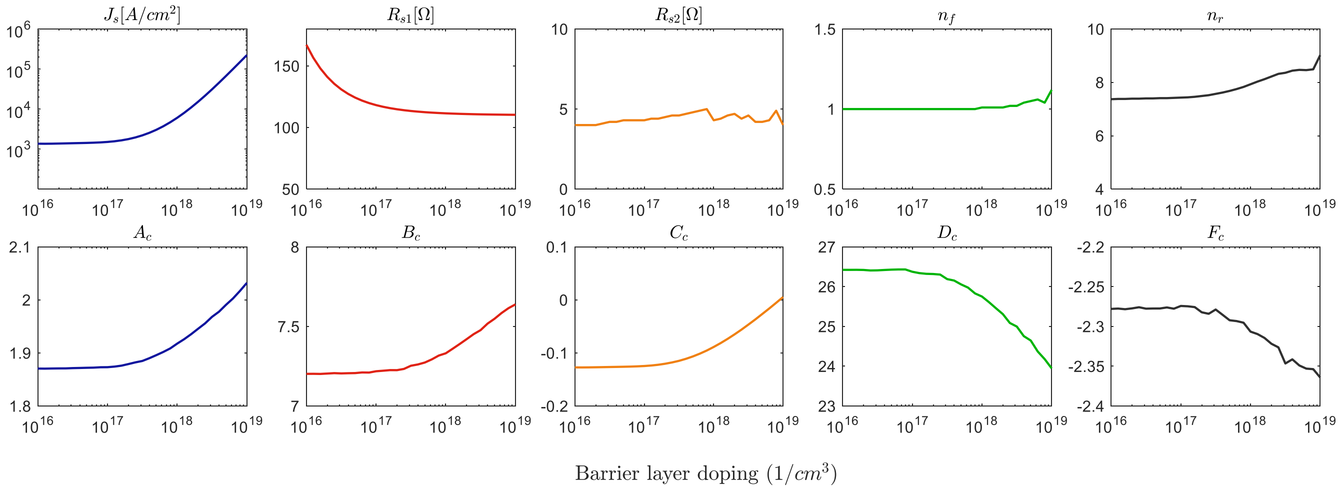

Supplementary Figure 2: Circuit model fitting parameters for IV (first row) and CV (second row) characteristics, corresponding to Equation 6 and Equation 8. The x-axis refers to barrier layer doping concentration in cm^{-3}.

Supplementary Results

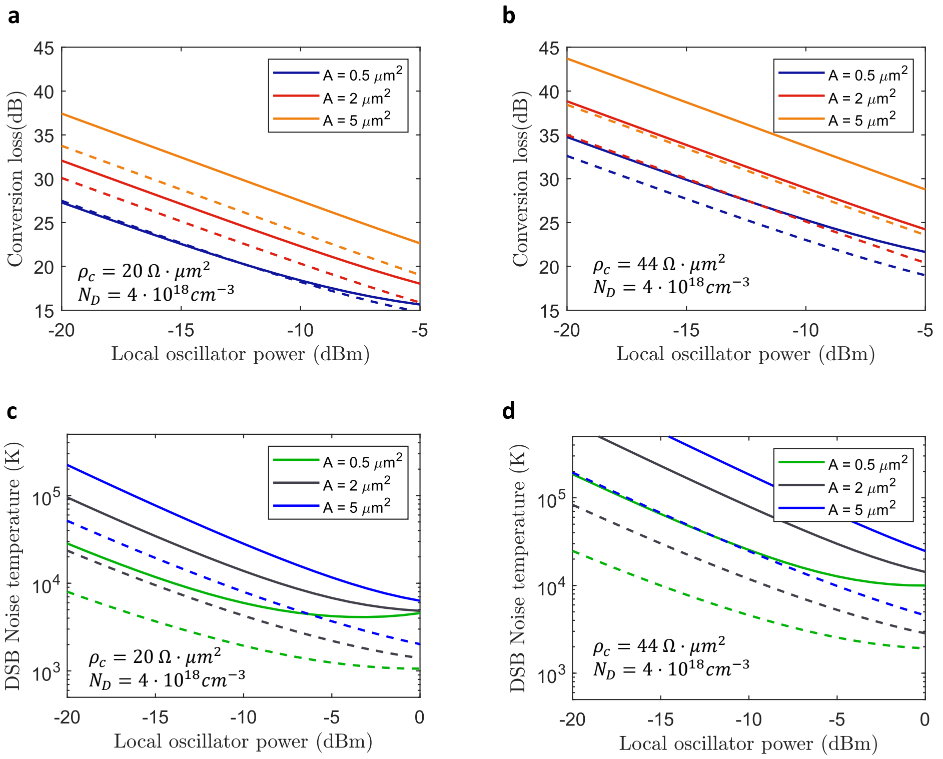

Supplementary Figure 3: 1D simulated conversion loss and noise temperature comparison for a doping of $4 \cdot 10^{18} cm^{-3}$ and RF frequency of 2 THz. Results are shown for contact resistivities of $44\Omega \cdot \mu m^2$ and $20\Omega \cdot \mu m^2$, with device areas of 0.5, 2, and 5 μm^2. Dotted lines refer to the case of using 4 barriers.

References

1. Adachi, S. *Physical Properties of III-V Semiconductor Compounds* (John Wiley & Sons, 1992).
2. Cavicchi, R. E., Lang, D. V., Gershoni, D., Sergent, A. M. & others. Admittance spectroscopy measurement of band offsets in strained layers of InxGa1-xAs grown on InP. *J. Phys. D Appl. Phys.* (1989).
3. Lang, D. V. *et al.* Measurement of heterojunction band offsets by admittance spectroscopy: InP/Ga0.47In0.53As. *Appl. Phys. Lett.* **50**, 736–738 (1987).
4. Forrest, S., Schmidt, P., Wilson, R. & Kaplan, M. Relationship between the conduction-band discontinuities and band-gap differences of InGaAsP/InP heterojunctions. *Appl. Phys. Lett.* **45**, 1199–1201 (1984).
5. Jain, S. & Roulston, D. A simple expression for band gap narrowing (BGN) in heavily doped si, ge, GaAs and GexSi1 x strained layers. *Solid State Electron.* **34**, 453–465 (1991).
6. Bugajski, M. & Lewandowski, W. Concentration-dependent absorption and photoluminescence of n-type InP. *J. Appl. Phys.* **57**, 521–530 (1985).
7. Li, J. C., Sokolich, M., Hussain, T. & Asbeck, P. M. Physical modeling of degenerately doped compound semiconductors for high-performance HBT design. *Solid State Electron.* **50**, 1440–1449 (2006).
8. Lopez-Gonzalez, J. M. & Prat, L. The importance of bandgap narrowing distribution between the conduction and valence bands in abrupt HBTs. *IEEE Trans. Electron Devices* **44**, 1046–1051 (1997).
9. Metzger, W. *et al.* Effective electron mass and plasma filter characterization of n-type InGaAs and InAsP. *J. Appl. Phys.* **92**, 3524–3529 (2002).
10. Sotoodeh, M., Khalid, A. H. & Rezazadeh, A. A. Empirical low-field mobility model for III–V compounds applicable in device simulation codes. *J. Appl. Phys.* (2000).

11. Rideout, V. L. & Crowell, C. R. Effects of image force and tunneling on current transport in metal-semiconductor (schottky barrier) contacts. *Solid State Electron.* **13**, 993–1009 (1970).

12. Kleinsasser, A. *et al.* n+ InGaAs/nGaAs heterojunction schottky diodes with low barriers controlled by band offset and doping level. *J. Vac. Sci. Technol. B Microelectron. Nanometer Struct. Process. Meas. Phenom.* **3**, 1274–1279 (1985).

13. Rideout, V. L. A review of the theory and technology for ohmic contacts to group III–V compound semiconductors. *Solid State Electron.* **18**, 541–550 (1975).

14. Nadar, S. *et al.* High performance heterostructure low barrier diodes for sub-THz detection. *IEEE Transactions on Terahertz Sci. Technol.* **7**, 780–788 (2017).
